# Patient side cost and its predictors for cervical cancer in Ethiopia: a cross sectional hospital based study

**DOI:** 10.1186/1471-2407-13-69

**Published:** 2013-02-08

**Authors:** Alemayehu Hailu, Damen Haile Mariam

**Affiliations:** 1 School of Public Health, Addis Ababa University, P.O.Box 9086, Addis Ababa, Ethiopia

**Keywords:** Cost of illness, Cervical cancer, Ethiopia, Human papilloma virus

## Abstract

**Background:**

Cervical cancer is a leading cause of death from cancer among women in low-resource settings, affecting women at a time of life when they are critical to social and economic stability. In addition, the economic burden is important for policy formulation. The aim of this study is to estimate patient side cost and to determine predictors of its variation for the treatment of cervical cancer.

**Methods:**

Analytic cross sectional study involving 227 cervical cancer cases at Tikur Anbessa Hospital, Ethiopia was conducted. Cost estimation was based on patients' perspective and using the prevalence-based model as a time frame. Productivity losses were estimated from lost working days.

**Results:**

The mean outpatient cost per patient for cervical cancer was $407.2 (Median = $206.9). Direct outpatient cost (Mean = $334.2) takes the largest share compared with the indirect counterpart ($150). The outpatient cost for half of the respondent falls in a range between $93.7 and $478. The mean inpatient cost for hospitalized patients was $404.4. The average direct inpatient cost was $329 (74% medical costs and 26% non medical costs). The mean value for total inpatient cost for half of the respondents was in the range of $133.5 and $493.9. For every additional day of inpatient hospital stay, there is a daily incremental inpatient cost of $4.2.

**Conclusion:**

As has been found in other studies, our findings revealed that cervical cancer creates an immense financial burden on patients. Primary prevention measures, vaccination against HPV and screening, should be initiated and expanded to reduce morbidity from cervical cancer and subsequent costs in both human lives and money resources. Control of co-morbidity and complication should be emphasized during management of cervical cancer patients. Capacitating regional hospitals and provision of low cost or fee exemption schemes should be arranged and strengthened.

## Background

Cervical cancer is a disease in which the cells of the cervix become abnormal and start to grow uncontrollably, forming tumors. It is considered a disease of early and late middle age. Isolated cases are found to occur among young women, but incidence rates are seen to rise sharply from age 35 years [1]. Cervical cancer is a leading cause of death from cancer among women in low-resource settings, affecting women at a time of life when they are critical to social and economic stability [2].

Worldwide estimates in 2010 indicate that every year 493,243 women are diagnosed with cervical cancer and 273,505 die from the disease. The prevalence of cervical cancer in the developing world is 59.4 per 100,000 [3]. In Africa the estimates indicate that every year 78,897 women are diagnosed and 61,671 die from the disease [4]. According to the WHO estimates, in Ethiopia 7,600 are diagnosed with cervical cancer and roughly 6,000 women die of the disease each year [5].

Although there is no national cancer registry in Ethiopia, reports from a retrospective review of biopsy results have shown that it is the most prevalent cancer among women. For instance, among 243 cancer cases, cervical cancer accounted for 12.8% of all cancers and 65.9% of female genital tract cancers in Gondar, Northwest Ethiopia [6]. Similar studies in Addis Ababa and Yirgalem Hospital (Southern Ethiopia) have also shown that it accounted for 32% [7] and 25.8% [8] of female malignancies, respectively. A study done on 2,111 women attending hospitals and clinics in Addis Ababa has also reported the prevalence of invasive cancer to be 15.6/1000 of the studied population [9].

With the magnitude outlined above, the economic burden of cervical cancer is considerable and highlights the need for treatment and prevention options for this condition. According to a review research in the United States, annual direct medical costs associated with cervical cancer range from 300 to 400 million USD. With a wide range across studies for estimates of the annual direct medical costs associated with carcinoma in-situ (CIN) which range from 700 million USD to 2.3 billion USD [10]. In the United States, although the direct costs of cervical cancer are substantial, only 10% of all expenditures are due to invasive disease; more than two thirds of the total cost being attributable to screening and testing. Annual indirect costs resulting from lost productivity and loss of earnings due to premature death are also significant and are estimated to be higher than the direct costs [11].

According to a population-based study in Spain, during a four year period (1999–2002), the mean cost of hospitalization due to cervical cancer and carcinoma in situ is 3,098 Euro and 2,192 Euro respectively [12]. Another retrospective study done in Tunisia has shown that the direct medical care cost of cervical cancer as ranging from 431 to 4143 Euro [13].

In Ethiopia no study has been documented that calculated the cost of cervical cancer. As resources are very limited and the cost of medical care is rising, it is critical to have an understanding of the economic aspect of cervical cancer in order to develop and implement sound public health policies. Therefore, this is study was designed to estimate costs of treatment and to determine predictors for variations in cost of cervical cancer. Both direct cost and productivity costs to the patients were investigated. The results of this study can be used as a basis for a full cost-utility analysis of the prevention and treatment of cervical cancer in the future in Ethiopia.

## Methods

### Study area

The study was carried out in the Departments of Gynecology/Obstetrics and Oncology/Radiotherapy of Addis Ababa University Tikur Anbessa Specialized Teaching Hospital. Tikur Anbessa Hospital is the only central referral hospital and cancer treatment and diagnostic center in the country that provides services for patients from all over the country. Women make up about 73% of the total patients at the center, and cancer of the cervix is the most common disease comprising over one-third of all female patients treated. The Gynecology/Obstetrics Department also provides surgical treatment and evaluation and screening of new and referral cases of cervical cancer, among other services.

### Study design

This study is a cross-sectional quantitative study using a hospital based analytic design.

### Study population and sampling methods

Histologicaly confirmed and clinically staged 227 cervical cancer patients who were attending outpatient and inpatient services at Tikur Anbessa Hospital (Gyn-OPD (40), gynecology ward (23), oncology referral clinic (117), and oncology ward (47)) within the specified period of data collection time (December 2011) and who had started treatment at least 3 months before the date of data collection were included in the study. The consecutive sampling technique was used to select the study subjects.

For each specified departments, participant cases were sampled proportional to size of the sample had been provided. Ethical approval was secured from the Research and Ethics Committee of the School of Public Health. Written informed consent was obtained from each study participant.

### The research instruments

Structured closed-ended and partially open ended questionnaires were used. The first draft questionnaires were in English and then they were translated to Amharic and then back translated to English to check for consistency. The questionnaires incorporate: general socio-demographic variables, direct cost (consultation cost, investigations cost, medicine cost, travel cost, food on the way to clinic cost), indirect cost (opportunity cost of lost working time) and socioeconomic characteristic of households. A pre-prepared checklist was used to collect clinical data from patients’ records. Six female nurses who have previous data collection experience were deployed after two days of training. Two data collection supervisors were assigned. The questionnaires were administered using face to face interview. Review of patient records was also done by the data collector that interviewed the respective study participant. Pre-test of the data collection instruments was done in another hospital in Addis Ababa among 10 women respondents.

### Method of cost estimation

This cost of illness study was conducted from the perspectives of the patients. Micro-costing and bottom-up approach was employed in order to estimate direct cost of cervical cancer. Indirect cost was calculated in terms of productivity time losses. As a time frame, prevalence-based model was used. Costs were calculated for each patient for the 12 months preceding the day of interview. The direct costs that were estimated include the direct medical costs for laboratory, medication, and consultation. Direct non-medical cost during outpatient visits and hospital admissions were also included.

The indirect costs that were estimated were earnings lost because of travel to outpatient visits/inpatients hospital stay and those due to absences from work because of illness related to cervical cancer. Time foregone in seeking care and productive time lost was converted into indirect cost based on the daily wage rate and then multiplied by the number of working days lost. The daily wage rate for monthly paid patients was estimated by dividing their net monthly salary by 30 days. Daily wage rate for daily paid patients were calculated based on the women’s reported daily earnings. The indirect cost for unemployed, students and women who were not able to work due to physical or mental disability were not considered in the calculations [14].

Individual cost items were summed up to the categories of medical costs, non-medical costs, and lost income. All costs were measured in Ethiopian Birr and were converted into US Dollar using the prevailing exchange rate during the time of the study (1 US Dollar = Eth Birr 14.5). The purchasing power parity (PPP) conversion factor for the Ethiopian Birr during the data collection period was 0.3 [15].

### Data analysis

The data were entered, cleared and analyzed using SPSS Version 16 for Windows. Data analysis was performed using scores, frequencies and percentages. A variety of descriptive statistics such as mean, standard deviations, medians and inter-quartile ranges were calculated. Before proceeding to further analysis, the residuals and the data had been examined for the fulfillment of the following statistical assumptions: normality, sensitivity, multicollinearity, and hetroscedasticity. Multiple linear regressions using a forward stepwise selection procedure, was employed to identify the predictors of cost variability. In the regression model, independent variables with a P-value < 0.1 have been entered whereas only statistically significant (P<0.05) variables were included in the final model.

## Results

### Characteristics of the study participants

A total of 227 study participants were interviewed. The mean (SD) age was 48.7 [12] years. The total numbers of participants in the age range of 18–64 years were 204 (89.9%). More than half of the study participants were married (52.4%), illiterate (54.6%), and housewives (52%). The majority (54.6%) of the respondents were from large households of greater than 6 individuals. Monthly mean household income was Birr 589 ($40.62) and the median was Birr 500 ($34.48).

About a quarter (26.4%) of the respondents reside less than 50 Km away from the Hospital. The rest (73.6%) of the patients need to travel more than 100 km in order to arrive at the Hospital from their place of residence. Mean (SD) distance of the study participants’ residence from the Hospital was 299.2 (239) km and the median distance was 300 km. Sixteen (7.1%) of the patients came from places that are more than 700 km away from the Hospital.

As illustrated in Table 1, cervical cancer stage was rated according to a staging scheme developed by the International Federation of Gynecology and Obstetrics [17]. Vast majority (46%) of the study participants were diagnosed at stage II followed by stage III (40%). Only 15 (7%) were diagnosed in the first stage of the cancer. Seventeen (7%) of the study participants were diagnosed at stage IV (see Table 1).

**Table 1:** Socio-demographic and other characteristics of the study subjects (*Birr 14.5=$1)

**Characteristics**	**n (%)**	**Characteristics**	**n (%)**
***Age Group***		***Occupation***	
15-24	4 (1.8)	House wife	118 (52.0)
25-34	21 (9.3)	Farmer	45 (19.8)
35-44	48 (21.1)	Government employee	13 (5.7)
45-54	75 (33.0)	Merchant	13 (5.7)
55-64	58 (25.5)	Private employed	12 (5.3)
64+	21 (9.3)	Daily laborer	8 (3.5)
***Ethnicity***		Retired	6 (2.6)
Tigray	24 (10.6)	Other	12 (5.2)
Amhara	104 (45.8)	***Family size***	
Oromo	64 (28.2)	1-2	22 (9.7)
Gurage	18 (7.9)	3-5	81 (35.7)
Others	17 (7.5)	6-8	24 (10.6)
***Marital Status***		9-11	70 (30.8)
Single	20 (8.8)	11+	30 (13.2)
Married	119 (52.4)	Monthly income in Birr*	
Widowed	57 (25.1)	1 - 299	32 (17.9)
Separated	17 (7.5)	300 - 599	78 (43.6)
Divorced	14 (6.2)	600 - 899	35 (19.6)
***Educational Status***		900 - 1199	16 (8.9)
Illiterate	124 (54.6)	1200+	18 (9.4)
Only read & write	44 (19.4)	***Distance from TAH in km***	
Grade 1-4	17 (7.5)	<50	60 (26.4)
Grade 5-8	18 (7.9)	51-300	76 (33.5)
Grade 9-10/12	22 (9.7)	301-500	48 (21.1)
College & above	2 (0.9)	501-700	27 (11.9)
***Religion***		700+	16 (7.1)
Orthodox	148 (65.0)	***Stage at diagnosis***	
Muslim	44 (19.0)	Stage I	15 (7.6)
Protestant	31 (14.0)	Stage II	104 (46.8)
Catholic	2 (1.0)	Stage III	91 (40.1)
Other	2 (1.0)	Stage IV	17 (7.5)

## Outpatient costs

### Direct cost

Among a total of 227 participants, 207 (91.2%) have visited at least one of the providers at least once. The rest 20 (8.8%) came directly to the Tikur Anbessa Hospital. Regarding the direct outpatient cost incurred at the Tikur Anbessa Hospital (Table 2), mean cost for consultation with a physician, investigations and medicines were estimated to be Birr 33.2 ($2.29), Birr 401.0 ($27.65) and Birr 568.7 ($39.22) respectively. From the total direct cost, the cost of medicine represents the largest share (48.4%), followed by investigations cost (34.1%).

**Table 2:** Direct outpatient cost in Birr, Addis Ababa, Ethiopia 2011 (*Birr 14.5=$1)

**Costs category**	**Mean (%)**	**Median**	**SD**	**(IQR)**
Before first visiting Tikur Anbessa Hospital				
*Direct medical cost*	*1002.9*	*403*	*2021.6*	*(1030, 100)*
Consultation Fee	33.2 (2.8)	20	47.6	(50, 25)
Investigations Fee	401 (34.1)	175	849.1	(500, 50)
Cost Of Drugs	568.7 (48.4)	100	1514.0	(450, 0)
*Direct non-medical cost*	*172.8*	*30*	*1329.6*	*(115, 4)*
Transportation Fee	81.6 (6.9)	25	147.8	(114, 3)
Cost for Other expenses	91.1 (7.8)	-	1261.3	(0, 0)
*Total direct cost*	*1175.7*	*475*	*2716.6*	*(1150, 150)*
At Tikur Anbessa Hospital				
*Direct medical cost (n=171)*	*3148.4*	*1280*	*5140.1*	*(3530, 445)*
Consultation Fee	111.2 (4)	30	596.9	(30, 5)
Investigations Fee*	1056.1 (34)	360	2357	(1000, 200)
Cost Of Drugs¥	1981.1 (63)	600	3796.7	(2000, 150)
*Direct non-medical cost*	*3568.6*	*1320*	*7828.2*	*(2995, 379)*
Transportation Fee (n=215)	117.3	70	304.1	(120, 10)
Non-prescribed remedies (n=206)	2816.6	600	7614.4	(2000, 100)
For food (n=212)	527.3	200	1143.3	(550,70)
*Total direct cost*	*7060.1*	*3425*	*10471.9*	*(7664, 1204)*

* Investigations include laboratory tests ultrasound, pathologic examination, x-ray and any other diagnostic procedures.

¥ Drugs include all medication prescribed by the physician or any other medication taken in relation with the problems of cervical cancer.

Direct cost for non-medical reasons takes the lowest share (14.7%) of the total cost. The mean cost for transportation was Birr 81.6 ($5.63). Expenditures for other items related with the illness (food, drink, bedroom, and non-prescribed drugs) amounted to Birr 91.1 ($6.28), which is 7.8% of the total direct cost.

The average outpatient direct cost per patient was Birr 7,060.1 ($486.91). The largest component was direct non-medical cost (mean = 3,568.6 ($246.11) and median = 1,320 ($91.03)). Mean expenses for non-prescribed remedies [Birr 2,816.6 ($194.25)] takes the largest share of non-medical direct costs followed by expenses for food [Birr 527.3 ($36.36)]. The mean cost of transportation fee was Birr 117.3 ($8.09).

The total direct medical cost for the 171 study participants was Birr 538,379 ($37129. 59). The average direct medical cost was Birr 3,148.4 ($217.13) (median = 1,280 and SD = 5,140.1). The mean cost for physician consultation, investigations and medicines came to Birr 111.29 ($7.66) (4%), 1,056.1 ($72.83) (34%) and 1,981.1 ($136.63) (63%), respectively.

### Indirect cost

On average, each of the cervical cancer cases had the illness for about nine months before first coming to the Hospital. But great variation was seen among the cases regarding the number of ill days with minimum of five days up to eight years (mean (SD) = 269.4 (317.4) days). The median duration of illness was 180 days. During the time interval between the dates of onset of symptoms to appearing at the Hospital, mean working days of 155.5 were lost (with a median of 2 months). During this time, the average loss of productivity amounted to Birr 1,228 ($84.69).

The time for single trip to reach health services varies from minimum of twelve minutes to maximum of ninety six hours (with mean (SD) and median of 5.7 [9] and 3 hours respectively.

The median duration of stay as an outpatient for the study participants was 180 days. In this duration, on average, the respondents had visited the Hospital outpatient 38 times. The mean loss of productivity was 119.4 working days. Total lost earning (indirect cost) during this time was Birr 273,195 ($18841. 03) (Median = 1,774 ($122.34)) (see Table 3).

**Table 3:** Indirect outpatient costs in Birr; Addis Ababa, Ethiopia 2011 (*Birr 14.5=$1)

**Costs category**	**Mean**	**Median**	**SD**
Before first visiting Tikur Anbessa Hospital			
Duration of complaint in days (n=207)	269.4	180.0	317.4
Days remained out of work (n=185)	155.5	60.0	334.6
Time for transportation	5.7	3.0	9.0
Amount of wages lost in Birr (n=145)	1228.9	180.0	2153.2
At Tikur Anbessa Hospital			
Duration of stay as outpatient in days (n=227)	374.1	180.0	826.3
Number of outpatient visits at TAH (n=220)	38.7	6.0	93.7
Days remained out of work in days (n=169)	119.4	30.0	206.2
Amount of wages lost in Birr (n=145)	1774.0	250.0	4618.9

### Inpatient/Hospitalization costs

#### Direct costs

From the total of 227 study participants, 122 (53.7%) were admitted and treated as inpatients in Tikur Anbessa Hospital or somewhere else for at least a day. Among the 122 hospitalized patients 98 (80%) had paid directly for their treatments. The treatment cost of the rest 20% was covered by other payers. The direct medical cost for the hospitalized study participants was Birr 347,321 ($23953. 17) (Mean = Birr 3,544.1 ($ (244.42) and Median = Birr 1,890 ($130.34)). The median cost for drugs was Birr 800 ($55.17), that for consultation was Birr 30 ($2.07), for investigation, it was Birr 500 ($34.48) and for medical supplies, it was Birr 120 ($8.28) (n=32). The total direct non-medical cost was Birr 121,408 ($8372. 96) (Median = Birr 800 ($55.17)). The median cost for non-prescribed remedies and for foods were Birr 300 ($20.69) and Birr 200 ($13.79) respectively (see Table 4).

**Table 4:** Direct and indirect inpatient cost of cervical cancer in Birr; Addis Ababa, Ethiopia 2011 (*Birr 14.5=$1)

**Cost category**	**Mean**	**Median**	**SD**	**(IQR)**
Direct costs				
*Direct medical cost (n=98)*	*3544.1*	*1890*	*5331.5*	*(3361, 722)*
Consultation	156.9	30	848.8	(50, 5)
Investigations	983.4	500	1650.5	(1000, 200)
Cost Of Drugs	2403.8	800	4231.3	(2500, 300)
Medical supplies (n= 32)	387.5	120	728.8	(500, 41)
*Direct non-medical cost*	*1152,0*	*800*	*1312.0*	*(1593, 200)*
Non prescribed remedies	676.6	300	1124.3	(962, 100)
For food	437.3	200	582.7	(600, 100)
*Total (n=93)*	*4771.0*	*2802*	*6356.0*	*(5418, 1574)*
Indirect costs				
Duration of inpatient stay in days (n=122)	34.0	16	42.2	(41, 7)
Days remained out of work (n=95)	41.6	20	55.2	(90, 1)
Amount of wages lost (n=50)	827.0	175	1871.0	(500, 100)

#### Indirect costs

With a total of 3.948 lost working days (Mean = 34 days, Median = 16 days), the total forgone earnings borne by the study participants because of cervical cancer due to hospital stay was Birr 41,353 ($2851. 93) [Mean = 827 ($57.03) and Median = 175 ($12.07)].

#### Predictors for variation in patient related cost

As provided in Table 5, the cost incurred by patients were computed across two broad distinct categories; outpatient (direct and indirect) and inpatient/ hospitalization (direct and indirect) cost for those who were ever admitted due to cervical cancer. To determine which socio-demographic/economic and clinical characteristics influenced cost, a multiple linear regression model was fitted to the cost data for both outpatient and inpatient separately. The final model includes seven variables, all of which were simultaneously significantly associated with outpatient cost of cervical cancer (P < 0.05): residency distance from the Hospital, the number of employed household members, number of facilities visited, occupation (farmer), companion, source of energy (animal dug) and residency (out of Addis Ababa).

**Table 5:** Outpatient and inpatient treatment cost in Birr summary; Addis Ababa, Ethiopia 2011(*Birr 14.5 = $1)

	**Outpatient cost**			**Inpatient cost**		
	**Direct (n=196)**	**Indirect (n=121)**	**Total (n=110)**	**Direct (n=93)**	**Indirect (n=50)**	**Total (n=110)**
Mean	4845.3	2173.7	5905.0	4771.0	827.0	5863.2
IQR	(4824, 651)	(3100,0)	(6933, 1359)	(5417, 1573)	(500, 100)	(7161, 1936)

Similarly, the prediction model for inpatient cost of cervical cancer was developed. The final model included three variables, all of which were simultaneously significantly associated with inpatient cost of cervical cancer (P < 0.05): duration of inpatient stay, co-morbidity and current stage of cervical cancer (Stage II). According to the model, longer duration of inpatient hospital stay and existence of co-morbidity were associated with higher inpatient cost. Patients currently (at the time of interview) at stage II incur additional Birr 4282.92 ($295.37) compared with patients in other stages (see Table 6).

**Table 6:** Multiple regression of variables on cost of cervical cancer; Addis Ababa, Ethiopia 2011 (*Birr 14.5=$1)

	**Unstandardized Beta (*B*)**	**t**	**p**	**Adjusted R**^**2**^
**Outpatient cost**				44.2%
(Constant)	3422.45	2.66	0.0095	
Residency distance from TAH (Km)	9.7	3.12	0.0025	
Number of employed household members	1434.26	3.88	0.0002	
Number of Facility visited $\left\{ \begin{array}{l} 0=otherwise\\ 1=if>1 \end{array}\right.$	3629.08	3.26	0.0016	
Occupation $\left\{ \begin{array}{l} 0=otherwise\\ 1=if\;Farmer \end{array}\right.$	7831.77	4.84	0.0001	
Source of Energy $\left\{ \begin{array}{l} 0=otherwise\\ 1=Animal\;dug \end{array}\right.$	15452.69	3.03	0.0033	
Companion $\left\{ \begin{array}{l} 0=had\;companion\\ 1=had\;no\;companion \end{array}\right.$	−4584.74	−3.03	0.0033	
Residency $\left\{ \begin{array}{l} 0=Addis\;Ababa\\ 1=out\;of\;Addis\;Ababa \end{array}\right.$	−5805.61	−3.25	0.0017	
**Inpatient cost**				46.6%
(Constant)	−415.96	−0.34	0.7321	
Duration of inpatient stay (day)	61.55	2.54	0.0147	
Co morbidity $\left\{ \begin{array}{l} 0=No\\ 1=Yes \end{array}\right.$	4274.68	2.73	0.0092	
Current stage $\left\{ \begin{array}{l} 0=otherwise\\ 1=Stage\;II \end{array}\right.$	4282.92	2.86	0.0066	

## Discussion

This study, in addition to confirming the costliness of cervical cancer as disclosed by other studies [17], has produced some important key findings related to the economics of cervical cancer that can be used as inputs for further economic evaluation.

The large differences between mean and median values of the various cost items indicated in this study could be due to the skewedness of the cost data. The standard deviations presented in this study are large, sometimes exceeding the means. Such a pattern is commonly observed when there is wide variation in treatment patterns among patients, even amongst those apparently exhibiting the same pattern of disease [18].

The mean age of the respondents was 48.7 years, which is an early menopausal period. Most (54.6%) of the respondents were illiterate followed by 23.2% of the respondents that hadn’t attended any formal schooling but can read and write. The vast majority (52%) were housewives. These findings show that the poor, marginalized and uneducated segments of the population are most affected by the disease. This may be useful information for policy actions aimed at addressing issues of inequity. These characteristics of the study participants were also consistent with other studies conducted in Ethiopia, and some other African and Latin American countries [9,17,19-21].

The median duration of illness was 180 days. Number of working days lost was also quite large (Median = 60 days). The delay may be due to low health seeking behavior, unawareness about available treatment options and lack of access to transportation or financial problems. The quality and structure of the referral linkage and mis-diagnosis due to lack of access to diagnostic instruments and skilled professionals at the peripheral health facilities may also be raised as reasons. Above all the status of women in decision making at household level may be one of the main contributing factors [22-26].

Any cost-of-illness study should always be viewed in the context of potential limitations. Some of the costs may be underestimated, some costs may be overestimated and some costs may be totally omitted. This cost of illness study is limited to the patient side cost, even though it would be more comprehensive if it includes other costs to the health system, health care provider, the family and to society at large. Intangible costs (pain, suffering, stigma and discrimination) were not also included due to difficulties in measurement [14]. The limitations of self-reported data must also be recognized in interpreting the findings of this study.

The outpatient cost for almost half of the respondent falls in a range between Birr 6,933 ($478) and Birr 1,359 ($93.7). The mean inpatient cost for hospitalized patients was Birr 5,863.2 ($404.4). The average direct inpatient cost was Birr 4,771 ($329) (74% medical costs and 26% non-medical costs). The mean value for total inpatient cost for nearly half of the respondents were in a range of Birr 7,161 ($493.9) and Birr 1,936 ($133.5). Even though it is difficult to compare the aforementioned findings with other studies done elsewhere it is quite clear that these cost estimates were big enough to be huge economic burden for the patients and their family members [13].

In terms of stages of illness, we found, the cost for stage I was lower compared with stage II and stage III. This finding was similar to findings from other study on cervical cancer [18,27]. The cost for stage IV was less compared with other lower stages. This could be due to the reason that cases at stage one might be recovered with minor procedures while other cases at other stages demand intensive diagnostic and therapeutic procedures as evidenced by the cervical cancer treatment protocol recommended by FIGO [16]. It may also be due to limited availability of treatment option for stage III and stage IV cervical cancer in Ethiopia so that the patients die quickly while a patient who has diagnosed at stage two tried all treatment options available.

This study showed that distance of patients’ residence from the Hospital, the number of employed household members, number of facilities visited, occupation (farmer), companion, source of energy used by patients household (animal dug) and residency (out of Addis Ababa) were significantly associated with costs. Some previous study reported in line with this finding [28,29].

There is no previous study which estimates the cost of cervical cancer in sub Saharan Africa. A comparison of our findings with those of other cost of illness studies of cervical cancer in other countries or other disease in Ethiopia would be of limited value because of the difference in the categories of cost, the methods, and the pattern of health services utilization. This could be the main weakness of any cost of illness study.

## Conclusion

 Cervical cancer creates an immense economic burden on patients and their families. The cost was dependent on the distance of patients’ residence from the hospital. Number of employed household members, number of facilities visited and the patient’s occupation was also a predictor of cost. Longer duration of inpatient hospital stay and existence of co-morbidity were associated with higher inpatient cost. Prevention of occurrence of cervical cancer shall be the prime focus in order to ultimately avoid the problem in all aspects including its economic burden. Primary prevention measures, vaccination against HPV and screening, should be initiated and expanded to reduce morbidity from cervical cancer and subsequent costs in both human lives and money resources. Once cancer develops, it needs to be controlled to avert the co-morbidities and complications, which can again significantly increase the cost of its management later. Capacitating regional hospitals, and provision of subsidized or exempted fee options for treatment and screening should be initiated and strengthened.

## Competing interests

The authors declare that they have no competing interests

## Authors’ contributions

AD has initiated the conception of the research idea. Both AH and DH participated in the writing of the proposal, design of the study, analysis of the data, interpretation, and manuscript preparation equally. Both authors read and approved the final manuscript.

## Pre-publication history

The pre-publication history for this paper can be accessed here:

http://www.biomedcentral.com/1471-2407/13/69/prepub
